# Expression and activation of intracellular receptors TLR7, TLR8 and TLR9 in peripheral blood monocytes from HIV-infected patients

**Published:** 2013-06-30

**Authors:** Guillermo J Valencia Pacheco, Francisc Pinzón Herrera, Juan J Cruz López, Ligia del Carmen Vera Gamboa, Norma Pavía Ruiz, Adrián Santos Rivero, Saulo Sánchez Lugo, Fernando Puerto

**Affiliations:** a Universidad Autónoma de Yucatán. Mérida, Yucatán, Méx. E-mail: jav-ree@hotmail.com; b Research of the "Dr. Hideyo Noguchi" Regional Biomedical Research Center. Email: vgamboa@uady.mx; c Centro Ambulatorio para la Prevención y Atención del SIDA y otras ITS (CAPASITS). Servicios de Salud de Yucatán. Mérida, Yucatán, Méx. E-mail: adrisan@prodigy.net.mx

**Keywords:** Innate immunity, monocytes, toll-like receptors, HIV

## Abstract

**Introduction:**

TLR´s play a role in host defense in HIV infection recognizing the viral DNA or RNA. Their activation induces a signaling pathway that includes the proteins MyD88, IRAK4, TRAF6 and the transcription factor NF-kBp65.

**Objective:**

To determine the expression of TLR7, TLR8 and TLR9, and activation of its signaling pathway in monocytes from patients infected with HIV. Methods. Expression of TLR7, TLR8 and TLR9 was determined in monocytes from HIV-infected patients (n= 13) and control subjects (n= 13), which were activated with specific ligands. The expression of MyD88 and NF-kBp65 were determined by flow cytometry; IRAK4 and TRAF6 were studied by immunoblotting.

**Results:**

No statistical difference was found in the expression of TLR7, 8 and 9 in monocytes from patients compared to controls, but we observed the non-significant increased expression of TLR9 in patients. The activation showed no significant difference in the expression of MyD88 and NF-kBp65 in patients when compared to controls, but were decreased in stimulated cells over non-stimulated cells. IRAK4 and TRAF6 were not detected.

**Conclusions:**

No statistical difference was observed in the expression of intracellular TLRs, MyD88 and NFkBp65 in monocytes from patients compared to controls. This was probably due to effective antiretroviral therapy being received at the time of study entry. Additional studies are needed under controlled conditions that include infected patients with and without ARVT, responders and non-responders, and work with different cell populations.

## Abstract (es)

**Introducción:**

En la infección por VIH los TLR juega un papel en la defensa del huésped al reconocer el ADN o ARN viral. Su activación induce la vía de señalización que incluye las proteínas MyD88, IRAK4, TRAF6 y el factor de transcripción NF-kBp65.

**Objetivo:**

Determinar la expresión del TLR7, TLR8 y TLR9, y activación de su vía de señalización en monocitos de pacientes infectados con VIH.

**Métodos:**

Se determinó la expresión de TLR7, TLR8 y TLR9 en monocitos de pacientes infectados con VIH (n= 13) y sujetos control (n= 13), se activaron con ligandos específicos y se determinó la expresión de MyD88 y NF-kBp65 por citometría de flujo. IRAK4 y TRAF6 fueron estudiadas por inmunoelectrotransferencia.

**Resultados:**

No se observó diferencia estadística en la expresión de TLR7, 8 y 9 en los monocitos de pacientes con respecto a los controles, pero observamos aumento no significante del TLR9 en los pacientes. La activación no mostró diferencia significativa en la expresión de MyD88 y NF-kBp65 en pacientes con respecto a los controles, pero se encontraron disminuidas en células estimuladas con respecto a las no estimuladas. IRAK4 y TRAF6 no se detectaron.

**Conclusiones:**

No se observó diferencia en la expresión de los TLR, ni en la expresión de MyD88 y NFkBp65, en monocitos de pacientes con respecto a los controles probablemente debido a la terapia antirretroviral recibida al momento del estudio. Se sugieren estudios con pacientes con y sin TARV, respondedores y no respondedores, y trabajar con diferentes poblaciones celulares.

## Introduction

Acquired Immune Deficiency Syndrome (AIDS) is a disease of the immune system caused by the of human immune deficiency virus (HIV) characterized by the progressive destruction of cellular components, primarily CD4+ T cells, which facilitates infection by opportunistic pathogens and development of neoplastic processes that can lead to death if antiretroviral therapy (ARVT) is not received^1^. Various aspects of HIV-host cell interactions have been addressed, such as cell tropism, activation and expression of associated receptor and co-receptors, signaling mechanisms, cells involved in the spread of infection, synergisms or inhibitory mechanisms with cytokine and viral factors in replication of the virus itself for the purpose of finding effective alternative treatments^2^.

In the mid 90's, the involvement of innate immunity mechanisms in the facilitation and/or resistance to infection and replication of HIV was presented which mainly focused on the stimulation, activation and expression of toll-like receptors (TLR). TLRs played an important role in pathogen recognition and in the initiation of immune and inflammatory responses^3^. Stimulation of TLRs by microbial products leads to the activation of signaling pathways that result in the induction of antimicrobial genes and inflammatory cytokines; additionally, stimulation catalyzes the maturation of dendritic cells and results in the induction of co-stimulating molecules and in increased antigen presentation capacity^4^ ^,^ ^5^.

 Ten human TLRs have been identified which differ from one another in ligand specificity, patterns of expression and inductive genes^3^. TLRs may be classified by the site of their expression and intracellular (TLR3, TLR7, TLR8, TLR9) and surface location (TLR1, TLR2, TLR4, TLR5, TLR6, TLR10)^6^ ^,^ ^7^.

With respect to intracellular TLRs (TLR7, TLR8, and TLR9), they share a specific signaling pathway that is dependent upon MyD88 (Myeloid differentiation primary response gene 88). The signaling cascade is mediated through interaction of MyD88 with IRAK4 (Interleukin-1 receptor-associated kinase 1) and TRAF6 (TNF receptor-associated factor 6) to form the MyD88/IRAK1/IRAK4/TRAF6 complex. Subsequently, IRAK1 and TRAF6 dissociate from the receptor complex and interact with kinases IKKB (IκB kinases) resulting in the activation of NF-kB (nuclear factor kappa-light-chain-enhancer of activated B cells), permitting the expression of genes of pro-inflammatory cytokine and chemokines such as TNF-α, IL-6, IL-8 and IL-1β^4^ ^,^ ^7^. On the other hand, the transcription factor IRF-7 (Interferon regulatory factor 7) can bind to the MyD88/IRAK1/IRAK4 complex, and its activation is dependent upon TLR7/TLR8 and TLR9, requiring the TRAF3 (TNF receptor-associated factor 3) protein which joins IRAK1 and IKKα kinases to produce interferon alpha (IFN-α)^7^.

Viruses and some bacterial pathogens may have access to cytosol and intracelular compartments^4^. Regarding TLR7 and TLR8, in vitro studies have been conducted using cell lines of dendritic cells (DCs) and macrophages derived from human monocytes to attempt to understand the pathogenesis of HIV infection and its relationship after TLR stimulation. In most of the studies complex contrasting mechanisms have been observed, since in specific stages of infection they appear to contribute to viral replication and dissemination that may be favored by concomitant stimulation by other TLRs with microbial products (LPS) in the course of opportunistic infections. On the other hand, in other stages they seem to have antiviral effects^8^ ^,^ ^9^. Furthermore, it has been reported in a mouse model that TLR9 stimulation by synthetic motifs, CpG-ODN (CpG oligodeoxynucleotides), after immunization with viral proteins (gp120) can significantly increase the humoral and cellular immune response and cellular protection against HIV^10^.

TLR7 and TLR8 are expressed on B cells and monocytes while DC plasmacytoids (DCps) express only TLR7 and immature DCs (DC11c+) express only TLR8. DCs, monocytes and macrophages are HIV target cells as well as DC4 + T lymphocytes; the latter cell line does not express TLR7/8. The stimulation of DC, monocytes and macrophages through TLR7/8 leads to activation of transcription factors, such as NF-kB in the individual´s host cells^8^. NF-kB is a critical component for the transcription of the majority of immune response genes that involve the production of antiviral cytokines and pro-inflammatories; however, it activates transcription sites present on the long terminal repetitions (LTR) of HIV, inducing transactivation and viral replication ^8^ ^,^ ^11^ ^,^ ^12^ ^.^

TLR9 is expressed in DCps and B cells intracellularly^13^ but may also be expressed on the surface of subtypes of cell populations depending on the tissue of origin. Its expression has been reported in immature DCs, in peripheral blood monocytes and in lymphocytes in lymph node tissue (tonsils)^14^. It has been observed that under normal conditions, stimulation of TLR9 with CpG ODN directly activates DCps and B cells, and can do so indirectly on monocytes, macrophages and NK lymphocytes, inducing increased expression of IFN type I^10^.

There is little information known regarding the role of TLR9 in HIV infection. However, there is in vitro evidence indicating that stimulation of mouse spleen cells with CpG ODN induces transactivation of the TRL of HIV-1 promoting infection and disease. This suggests that these mechanisms can be synergized when there is a concomitant stimulation of TLR9 with CpG ODN and TLR4 with the LPS ligand^12^. Probably these favorable viral replication conditions are present in Gram negative opportunistic pathogens in patients with AIDS.

There is no information about the expression of intracellular TLR (TLR7, TLR8, and TLR9) in monocytes from HIV-infected patients, and it is unknown whether there are alterations in the signaling pathway used by these receptors. Most studies have used mouse models or cell lines and cultures mainly of mature DCs, DCps, and macrophages derived from human monocytes^4^ ^,^ ^8^ ^,^ ^9^ ^,^ ^10^ ^,^ ^15^ since these cells are the first line of defense for the immune system, are the first to be infected with HIV, and are responsible for spreading the infection to DC4 + T lymphocytes. To this point there are no reports of human studies that have evaluated the expression of intracellular TLR and the activation of its signaling pathways.

The objective of this work was to study the expression of TLR7, TLR8 and TLR9 intracellular receptors, and the activation of its signaling pathway in the monocyte population starting with peripheral blood mononuclear cells (PBMC) of HIV patients to contribute to the knowledge of involvement by innate immune mechanisms in the pathogenesis of the disease, supporting the search for possible therapeutic alternatives that involve these structural components.

## Materials and Methods

### Patients and healthy control.

Thirteen patients were studied who were diagnosed with HIV/AIDS and attended to at CAPASITS (Outpatient Center for the Prevention and Care of AIDS and Sexually Transmitted Infections) of O'Horan Augustín General Hospital of the Health Secretariat of Yucatan, Mexico, and 13 subjects included in the control group who agreed to participate and signed a written consent for the study. The control group was defined as apparently healthy individuals who were serologically negative to HIV infection. Patients and controls were matched by age and sex. The study was reviewed and approved by the ethics committee of Augustín O'Horan General Hospital.

### Isolation of peripheral blood mononuclear cells (PBMC)

Heparinized venous blood samples were obtained from patients and controls, and PBMC were isolated using the Ficoll Hypaque density-gradient centrifugation technique^16^. The cells were re-suspended in a complete medium (cultured medium of RPMI 1640 supplemented with 10% fetal bovine serum, 100 U/mL of penicillin, 100 μg/ml streptomycin, and 0.2 mM of L-Glutamine). Cell viability was determined by an inventory of a Neubauer chamber and staining with trypan blue. The PBMC were adjusted to a concentration of 1 x 10^6^ cells/mL in a complete medium.

### Activation tests to determine MyD88, NF-kBp65, IRAK4, and TRAF6

To study the signaling pathway of TLR, the PBMC were adjusted to 1 x 106 cells/mL in complete medium and specifically stimulated, or failed to, with the ligand for TLR7 (imiquimod (r-837 (IMG-2207)) or TLR9 (CpG-ODN 2006 B (IMG-2209H) (Imgenex Corp.), at 37° C during the indicated times (2 and 24 hours). The cells were then washed by centrifugation (1,000 rpm for 10 minutes). To identify MyD88 protein in activated and non-activated cells (2.5 - 5 x 10^5^ cells/mL), surface staining was first carried out to identify monocytes and afterwards intracellular staining using the kit IC-Flow (IMGENEX, Cat 10083K, San Diego, CA); the cells were first incubated with monoclonal human anti-MyD88 not marked (MAB29287 (RD Systems)) and then with secondary antibody anti-rat IgG labeled with FITC (F0104B (RD Systems)). To identify the transcription factor NF-kBp65, and after staining the monocyte population, intracellular staining was performed using monoclonal human anti-NF-kBp65 marked with PE (PS529 (BD PhosFlow).) Cells were analyzed in the flow cytometer FACSCalibur (Becton Dickinson) using CellQuest software.

The cytosolic fraction was obtained from stimulated and non-stimulated cells by means of the nuclear extraction kit (Nuclear Extraction kit. 110081 K, IMGENEX Corp.) following the supplier's instructions. To determine IRAK4 and TRAF6 molecules, the cytosolic fraction was subjected to electrophoresis in a 14% polyacrylamide gel (SDS-PAGE) and proteins transferred to nitrocellulose membranes. After blocking with 5% low fat milk in a solution of TBS-T/(200 mM Tris HCl pH 7.5, 1.5 M NaCl and 0.05% Tween 20), the membranes were incubated with corresponding polyclonal antibodies to detect corresponding TRAF6 (IMG -536) and IRAK4 (IMG-441) proteins. The membranes were then washed and incubated with the secondary antibody conjugate with a radish peroxidase (Cat. 20301, IMGENEX Corp.). The protein bands were visualized by an immunoenzymatic reaction using TMB substrate (T0565, Sigma-Aldrich) following the supplier's directions.

### Statistical analysis

The statistical analysis was performed using the GraphPad Prism5(r) program. The percentages of expression and mean fluorescent intensity (MFI) of TLR in monocytes were evaluated following criteria of normality; they were analyzed using the parametric Student t test if they fulfilled the stated criteria, and the nonparametric Mann-Whitney U in other cases. The correlation of TLR expression with the molecules of the signaling pathway was determined by means of the Spearman rank correlation. Data are presented as means ± SD and a significant difference was considered at the *p* <0.05 level.

## Results

### TLR expression in monocytes

**Figure 1:**
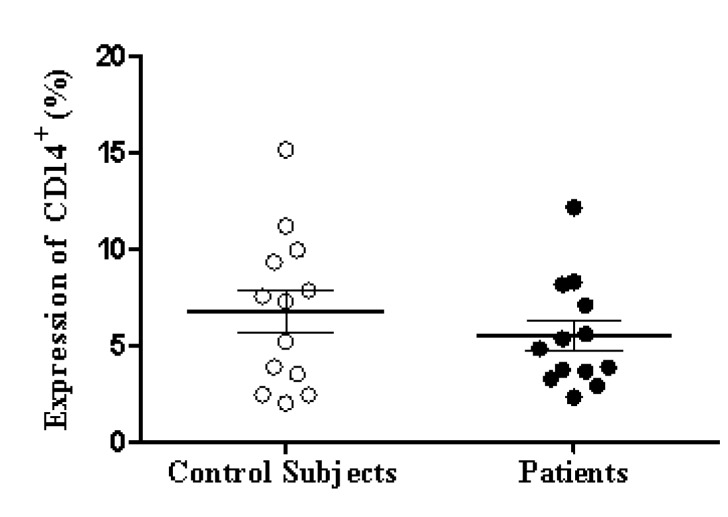
Expression of monocytes The PBMC of patients (n= 13) and control subjects (n= 13) were immunostained for DC14 and analyzed by flow cytometry as described in the methodology section. Data are presented as the mean and standard deviation of the percentage of positive DC14 cells. No significant differences between patient and control group (p > 0.05) were found. Statistical tests used: bilateral paired t and Mann-Whitney.

**Figure 2.**
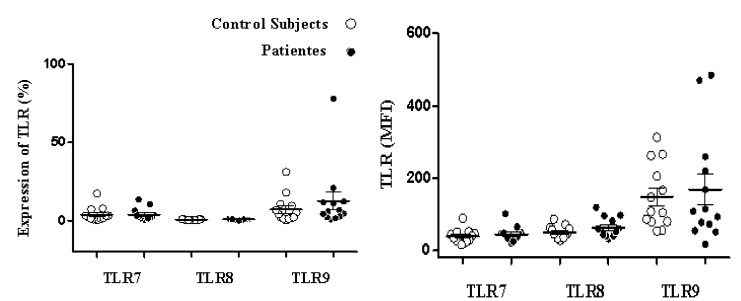
Expression of intracellular TLR in peripheral blood monocytes The PBMC (n= 13) of patients and control subjects (n= 13) were immune-stained for DC14, TLR7, TLR8, TLR9, and analyzed by flow cytometry, as described in material and methods section. Data are the mean and standard deviation of the percentage of DC14 positive cells expressing TLR7, TLR8, and TLR9 (A) and the mean fluorescent intensity (MFI) of TLRs (B). No significant difference between patients and control groups were found (p > 0.05). Statistical tests used; bilateral paired t and Mann-Whitney.

The percentage of expression of TLR7, TLR8 and TLR9 was determined for the monocyte populations of 13 patients diagnosed with HIV and 13 apparently healthy subjects used as controls. Clinical data for the patients is shown in Table 1. First, the percentage of monocytes between patients and controls was determined, and a decrease in the population of monocytes with the patient group was observed in comparison with the control group, but it was not statistically significant (*p* = 0.1939) (Fig. 1). The percentage of TLR7, TLR8 and TLR9 expression in the monocyte population showed no significant differences between the patient and control groups (Table 2). However, we observed a non-significant increase in the expression of TLR9 in the patient group as compared to the control group (Fig. 2 A and B).

**Table 1:**
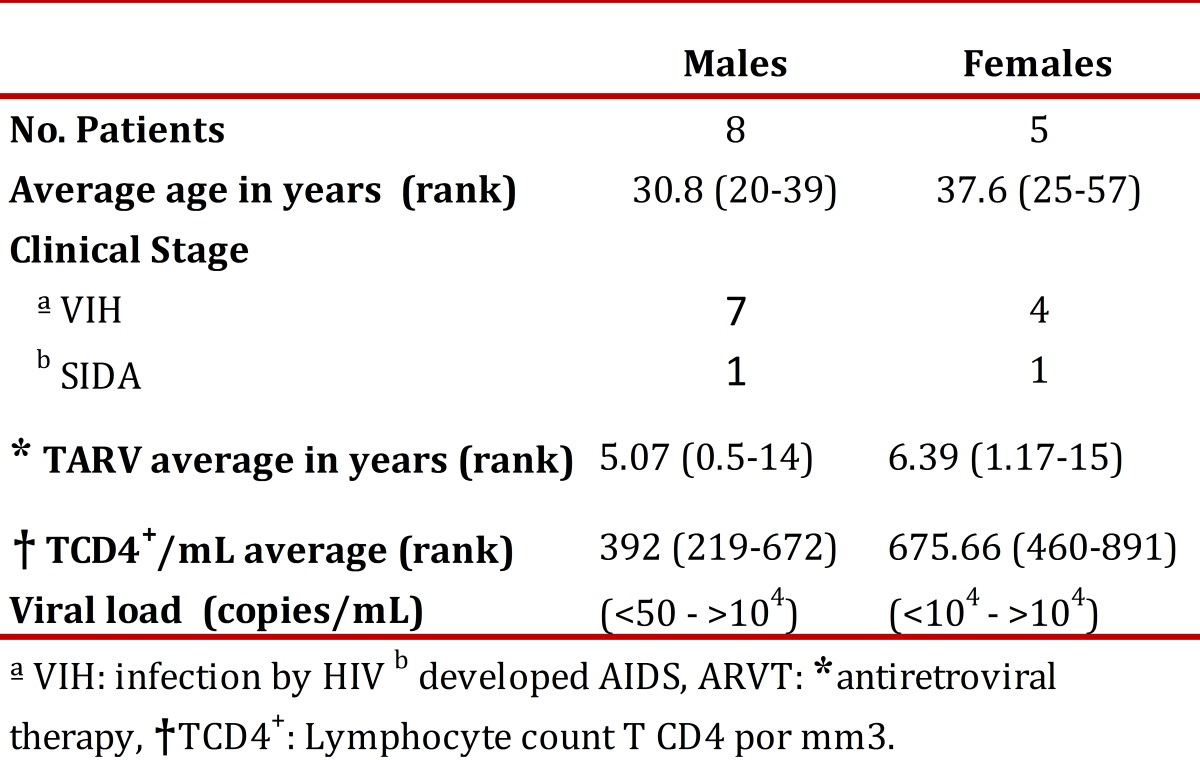
Clinical data of patients infected by HIV .

**Table 2:**
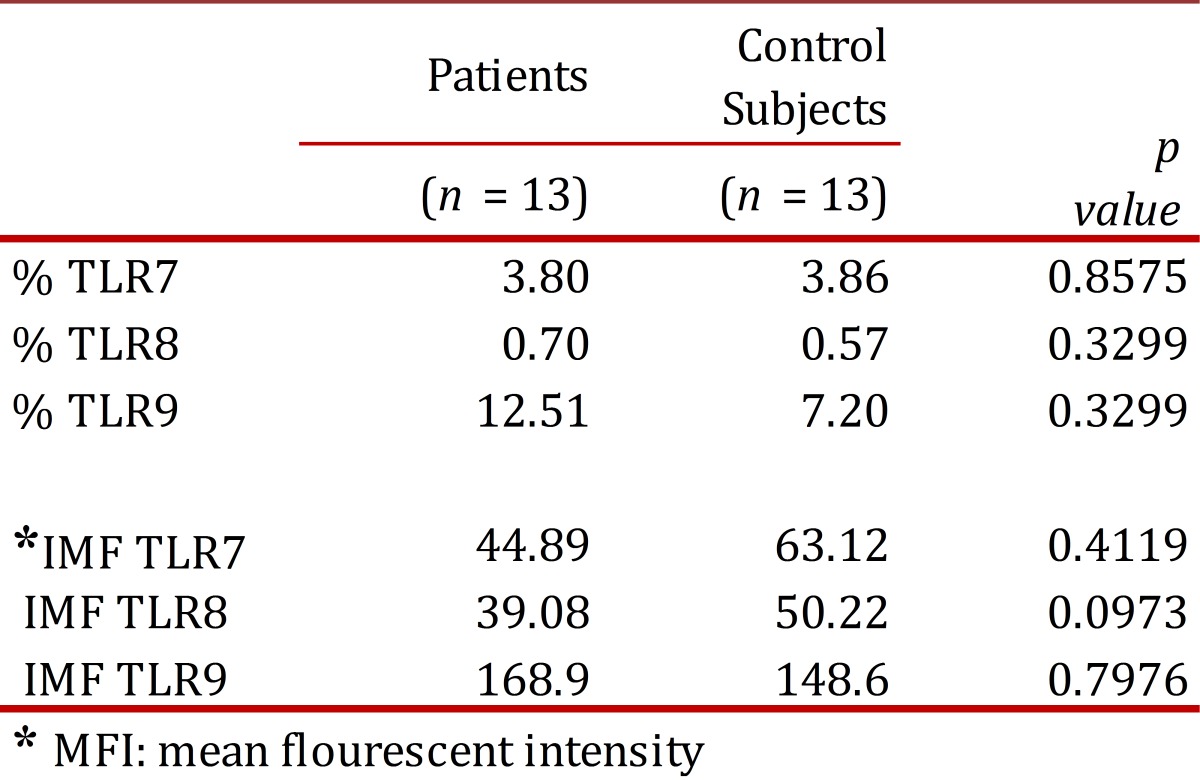
Percentage of expression (%) and mean fluorescent intensity (MFI) of TLR7, TLR8 y TLR9 in monocytes from peripheral blood for patients and control subjects.

### Expression of MyD88 and NF-kBp65

**Figure 3:**
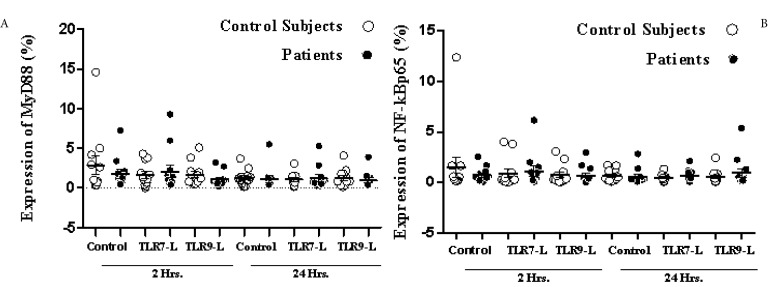
Expression of MyD88 (A) and NF-kBp65 (B) in peripheral blood monocytes. The PBMC (n = 12) of patients and control subjects (n = 12) were immune-stained for DC14, MyD88 and NF-kBp65 and analyzed by flow cytometry, as described in material and methods section. Data presented are the mean and standard deviation of the percentage of DC14 positive cells expressing MyD88 (A), and NF-kBp65 (B). No significant difference between patients and control groups were found (p> 0.05). Statistical tests used: bilateral paired t and Mann-Whitney.

Proteins were identified in the monocyte population for 12 patients with their respective controls due to the poor performance of mononuclear cells. There was no significant difference observed in the percentage of MyD88 expression between the patient and control group (*p* > .05). With respect to the time of stimulation, there was also no significant difference in protein expression between 2 and 24 hours in either of the study groups (*p* > 0.05). However, a slight decrease was observed in the percentage of of MyD88 expression at 2 hours of stimulation with the TLR9 ligand in the patient group with respect to non-stimulated cells. The TLR7 ligand showed no increase or decrease of MyD88 in stimulated and non-stimulated cells in the patient group. There was no increase or decrease in the expression of MyD88 between the two groups at 24 hours (Fig. 3)

The percentage of NF-kBp65 expression showed a behavior similar to that observed with MyD88. Low expression NF-kBp65 was observed in both study groups with respect to the stimulation time. The control group showed a slight, insignificant increase in the expression of NF-kBp65 at 2 hrs in non-stimulated cells when compared to non-stimulated cells of the patient group (*p* > 0.05). The IRAK-4 and TRAF6 proteins were not detected in the cytosolic fractions obtained from non-stimulated and stimulated cells with the specific ligand for TLR7 and TLR9.

The correlation between the percentage of expression of the TLRs (TLR7, TLR8, TLR9) with MyD88 and NF-kBp65, respectively, was determined at baseline (non-stimulated cells) in order to establish the possible association between the expression of the receptors with signaling molecules (Table 3). None of the TLR showed significant correlations with the proteins (*p* > 0.05).

**Table 3:**
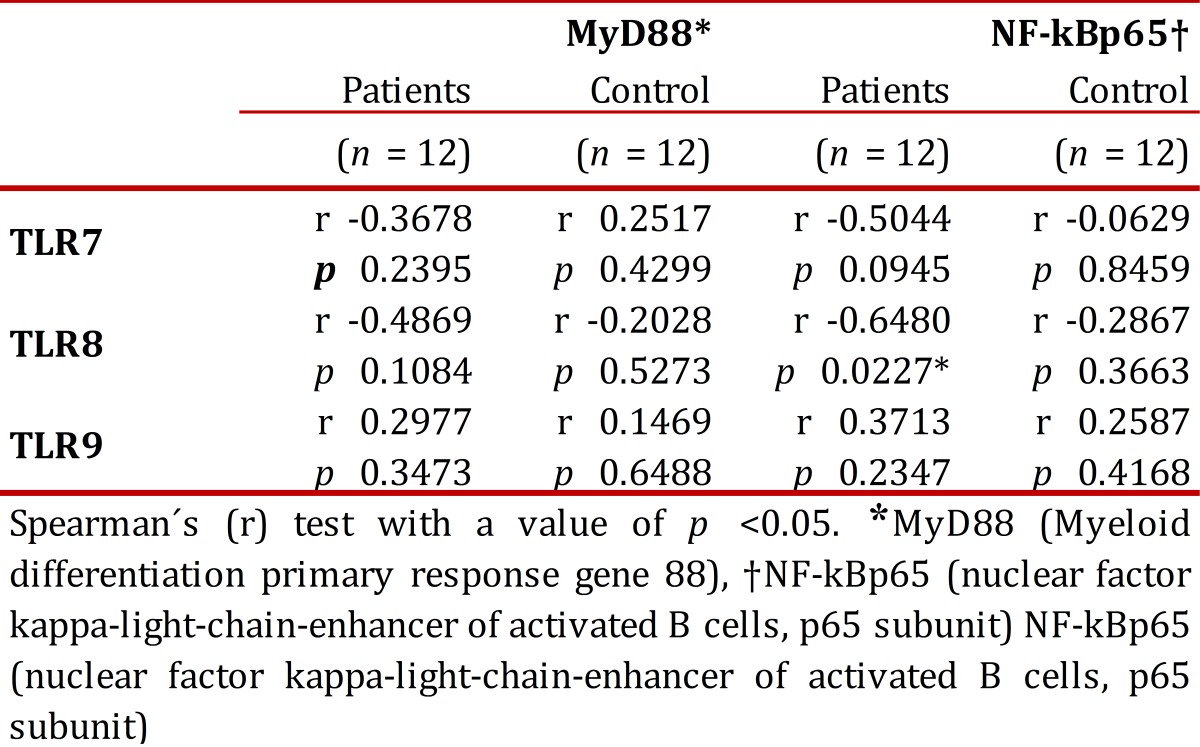
Correlational analysis of the expression of TLRs (TLR7, TLR8, TLR9) with the expression of MyD88 and NF-kBp65 on the baseline state of peripheral blood monocytes from patients and control subjects.

## Discussion

This is the first report which describes the expression of intracellular receptors TLR7, TLR8, TLR9 and the activation of its signaling pathway in the peripheral blood monocyte population of patients infected with HIV by means of flow cytometry and may be considered a pilot study.

First, we determined the percentage of monocytes (DC14+) in patients and controls, so that the results obtained from the determination of TLR expressions and activation of the signaling pathway were not influenced by an alteration in the population of interest. Expression values of monocytes between the groups in the study showed no significant difference; however, the patient group showed a decrease in these cells. At least two subpopulations of monocytes have been reported in HIV infection: a population that expresses high levels of DC14 and low levels of DC16 (DC14high DC16low), and another which expresses low levels of DC14 and high levels of DC16 (DC14^low^ DC16^high)^.

This indicates that the HIV infection appears to be associated with the expansion of the population of monocytes DC14low DC16high^16^. Moreover, it has been described that ARVT normalizes the production cell progenitors, induces change in T cell subtypes, reverses the defects of DC4+ cells, and restores the production of cytokines, such as IL-2, and the reactivation of lymphocytes^17^. This indicates that ARVT has an important effect on restoring the immune response^18^ ^,^ ^19^.In this regard, the data obtained in this study suggests that the decreased, non-significant expression of monocytes in patients may be due to the ARVT administered promoting the appearance of the DC14low DC16high monocyte populations. Taking clinical data into account, we can suggest that in our patients receiving ARVT the subpopulation of monocytes present is DC14high DC16low; however, these were not identified, which raises the need for further studies to establish their role in the infection.

Lester *et al* ^20^ reported that by means of PCR multiple TLRs are increased in untreated, chronic HIV infection, and that this effect is reversed when viral replication is inhibited after ARVT, suggesting that viral replication leads to increased in vivo expression of TLR. However, no difference was found in the expression of TLR9 in patients with chronic infections and AIDS, and controls, but increased expression of TLR7 and TLR8 was shown.

On the other hand, Scagnolari *et al *.^21^ did not observe a difference in TLR7 (mRNA) expression levels between controls and patients infected with HIV. However, those responding to ARVT had an elevated tendency in TLR7 expression in MNC of infected patients, responders or non-responders to ARVT. Similarly, a significant difference was not observed in the expression of TLR9 (mRNA) between the infected group, responders to ARVT, and the control group, but a significant decreased expression of TLR9 in infected patients responding to ARVT (in whom treatment was discontinued), and non-responders was observed.

The results obtained in this study seem to agree with the data from Scagnolari *et al* and suggest that the non-significant, increased percentage of TLR9 expression observed in the patient group was probably due to the antiretroviral therapy they were receiving at the time of study entry. In spite of the data that suggest that ARVT can reverse increased TLR expression associated with viral replication, additional studies are needed to investigate the percentage of TLR9 expression, and other TLRs, in infected patients with and without ARVT, responders and non-responders to ARVT. Moreover, we must also take into account the intrinsic characteristics of the infected patients that receive ARVT, such as the presence of genetic polymorphisms of resistance to the progression of the AIDS infection, and the capacity to respond to different ARVT^22^ ^-^ ^24^.

On the other hand, Scagnolari *et al *reported that the average time of ARVT in responding patients was 7 years (3 -13 years), which is very similar to our study group: 6 years (0.5 to 15 years). Regarding the viral load (VL), decreased TLR7 expression was reported (not at significant levels) along with that of TLR9 (significant) in patients with VL <5 log cop/mL compared with those patients with high levels of VL. Our group of patients showed a VL between <50 cop/mL- <4 log cop/mL (Table 1). These data show a consistency with those reported by Scagnolari *et al *. and suggest that the non-significant percentage of TLR7, TLR8 and TLR9 expression in HIV infected patients is due to the low levels of VL as a result of ARVT being administered.In spite of the data and studies, it is important to note that few studies involve intracellular TLR expression in cell populations of HIV infected patients. Moreover, PBMC have been studied but not in the monocyte population, and they have determined the levels of mRNA expression of TLR by RT-PCR. It should be noted that PBMC represents a variety of cell types with different patterns of expression of receptors that respond to different stimuli or viral ligands^25^. Kadowaki *et al* ^26^ and Hornung *et al* ^12^ reported the pattern of TLR expression by RT-PCR in the monocyte population of apparently healthy individuals, observing high expression levels of TLR1,^1^ ^-^ ^4^a moderate expression of TLR8, and a weak expression of TLR7 and TLR9. The authors report that the weak expression of TLR9 in monocytes probably did not allow for an appropriate response to the specific ligand of CpG-ODN. In contrast, Gorden *et al* ^27^ reported that monocytes, monocyte-derived DC, and mDC respond to TLR8 ligands and to a lesser extent to the TLR7 ligands.

Regarding the TLR signaling pathway, we did not observe a significant difference in the expression of MyD88 and NF-kBp65 in non-stimulated and stimulated PBMC with specific ligands between patients and controls. Lester *et al *reported an increase in mRNA expression of TLR7 and TLR8 in subjects infected without any ARVT, which could be correlated with an increase in the induction and expression of proteins involved in the signaling pathway of these receptors. The results obtained of the expression of NF-kBp65 and MyD88 suggest that there are no alterations in the signaling pathway of these receptors, probably due to the therapy received, leading to decreased expression of IRAK-4 and TRAF6 proteins through the restoring effect of ARVT. However, additional studies are needed to determine the effect of antiretroviral therapy on the expression and activation of the signaling pathway of intracellular TLR in HIV-infected patients. The correlational analysis of TLR7, TLR8, and TLR9 with MyD88, and NF-kBp65, respectively, shows a significant negative correlation between the TLR8 and NF-kBp65. This seems to indicate that the expression of TLR8 may be decreased as a function of the increase in expression of NF-kBp65, or vice versa. To give a better interpretation of this result, it is necessary to conduct studies under controlled conditions with larger samples of patients, with and without antiretroviral therapy, responders and non-responders, and the possibility of work with different cell populations to determine that the unaltered expression and activation of TLRs is due to the effectiveness of ARVT.
